# NIR-II fluorescence microscopic bioimaging for intrahepatic angiography and the early detection of *Echinococcus multilocularis* microlesions

**DOI:** 10.3389/fbioe.2023.1157852

**Published:** 2023-04-19

**Authors:** Nuernisha Alifu, Ting Yan, Jun Li, Lijun Zhu, Abudusalamu Aini, Siyiti Amuti, Juan Wu, Wenjing Qi, Gang Guo, Wenbao Zhang, Xueliang Zhang

**Affiliations:** ^1^ State Key Laboratory of Pathogenesis, Prevention and Treatment of High Incidence Diseases in Central Asia, School of Medical Engineering and Technology, Xinjiang Medical University, Urumqi, China; ^2^ State Key Laboratory of Causes and Prevention of High Morbidity in Central Asia, The First Affiliated Hospital/Institute of Clinical Medicine, Xinjiang Medical University, Urumqi, China; ^3^ Department of Human Anatomy, School of Basic Medical Sciences, Xinjiang Medical University, Urumqi, China

**Keywords:** near-infrared-II, hepatic alveolar echinococcosis, early diagnosis, fluorescence microscopic imaging, indocyanine green

## Abstract

Hepatic alveolar echinococcosis (HAE) is caused by the metacestode of *Echinococcus multilocularis*, which shows characteristics of malignant tumors with high mortality. However, traditional diagnostic imaging methods are still not sufficient for the recognition of HAE microlesions in the early stages. Near-infrared-II (900–1700 nm, NIR-II) fluorescence microscopic imaging (NIR-II-FMI) has shown great potential for biomedical detection. A novel type of negative target imaging method based on NIR-II-FMI with the assistance of indocyanine green (ICG) was explored. Then, NIR-II-FMI was applied to the early detection of HAE for the first time. The negative targeting NIR-II fluorescence imaging of HAE-infected mice at different stages with the assistance of ICG under 808 nm of laser irradiation was obtained. Especially, HAE microlesions at the early stage were detected clearly. Moreover, clear intrahepatic angiography was achieved under the same NIR-II-FMI system.

## 1 Introduction

Hepatic alveolar echinococcosis (HAE), which is known as “worm cancer,” is a parasitic disease caused by the metacestode of the dog/fox tapeworm *Echinococcus multilocularis* (*E. multilocularis*) (Qian and Zhou, 2018; Wen et al., 2019) and is distributed to most areas in the Northern Hemisphere (Li et al., 2015). HAE is a deadly disease with a mortality rate of more than 90% within 10–15 years without treatment (Mcmanus et al., 2012). The disability-adjusted life years (DALYs) for humans with HAE is 666,434 (Torgerson et al., 2010), indicating that this disease causes a heavy economic burden and has a lot of harm effects (Deplazes et al., 2017; Qian et al., 2019). The existing treatments for HAE mainly include radical resection combined with the long-term use of antiparasitic drugs, but the curative effect of these treatments is poor (Dezsényi et al., 2017). Most of the patients with HAE are at the late stage due to the difficulty in detecting the infection in the early stage (Wang et al., 2018; Chen et al., 2019). Hence, an early diagnosis, especially an imaging-based diagnosis, is urgently needed to improve the treatment efficiency for HAE (Bhutani and Kajal, 2018).

At present, the diagnosis of HAE mainly utilizes imaging techniques also considering the epidemiological history, clinical manifestations, and etiological diagnosis (Mihmanli et al., 2016). Imaging examination is mainly used to evaluate the location and size of HAE lesions in the liver. The imaging methods utilized in HAE diagnosis include ultrasound, computed tomography (CT), magnetic resonance imaging (MRI), and X-ray examination (Li et al., 2020). However, these imaging methods have their own advantages and limitations. For example, the resolution of ultrasound is not high enough for detecting *E. multilocularis* at an early stage (Tao et al., 2016). Additionally, serological methods lack specificity and show low sensitivity for detecting small metacestode lesions (Hemphill et al., 2014). The techniques for detecting small lesions of HAE are, thus, in great demand (Gottstein et al., 2015; Wen et al., 2019).

Fluorescence imaging technology has the advantages of high sensitivity, high spatial resolution, and non-invasiveness, which can help in the observation of biological structures and dynamic processes, accurately (Copeland and Aronson, 2015; Tamer et al., 2015). However, high-quality fluorescence imaging needs to overcome scattering from the bio-tissue. Thus, there is a great demand for long wavelength light in the optimized optical band to solve this problem. However, light in the visible (400–750 nm) and near-infrared (NIR, 750–900 nm) spectral regions, which are mainly utilized in traditional fluorescence imaging methods, still suffer from scattering and autofluorescence from the deep tissue (Abuseir et al., 2013; Wang et al., 2013; Nicolao et al., 2014; Giorgi et al., 2015). Compared with the visible and NIR-I light, the light in the near-infrared II (NIR-II, 900–1,700 nm) spectral region shows longer wavelengths with less scattering, which can improve the imaging depth and spatial resolution (Chen et al., 2014; Hong et al., 2017; Zhang et al., 2018; Feng et al., 2019). Moreover, NIR-II fluorescence imaging in deep tissues has exhibited very low autofluorescence, which is helpful for obtaining fluorescence bio-images with a high signal-to-noise ratio and contrast (Alifu et al., 2018; Wan et al., 2018; Fan et al., 2019; Yu et al., 2019). Thus, NIR-II fluorescence imaging technology becomes a powerful imaging method that can achieve clear images of microlesions with fine structures (Lin et al., 2018; Shou et al., 2018; Wu et al., 2020), especially NIR-II fluorescence microscopic imaging (NIR-II-FMI) technology, which combines NIR-II fluorescence imaging with microscopic fluorescence imaging; it can help in obtaining precise and high-quality images of the fine structure in bio-samples (Qi et al., 2018; Zebibula et al., 2018; Yang et al., 2022) reported an I-PVA@PDA focal necrosis of hepatocytes with necrotic cell fragments and inflammatory cell infiltration in liver tissue with microspheres, which has great potential in fluorescence imaging. Jessica et al. (Carr et al., 2018) utilized indocyanine green (ICG) as a fluorescence agent for *in vivo* NIR-II fluorescence imaging. In addition, liposomes serve as good drug delivery systems and have been utilized to encapsulate ICG for NIR fluorescence imaging and photothermal therapy (Xiong et al., 2022). However, as far as we know, there has been no report of the negative target imaging of NIR-II fluorescence imaging with the assistance of free ICG in the early detection of HAE and intrahepatic angiography until now. Zhang et al. (2023) found that the modification of bovine serum albumin can greatly improve the biocompatibility of the MPB platform and alleviate acute liver injury caused by oxidative stress in the treatment of oxidative stress-induced acute liver injury using the molybdenum-based nanoplatform with multi-enzyme simulation ability.

In this study, we set up an NIR-II-FMI system with an 808 nm laser as the excitation source. We further utilized clinical ICG as an NIR-II fluorescence probe (Carr et al., 2018) and analyzed the NIR-II fluorescence properties of the clinical ICG *in vitro* and liver cells. Then, we established different stages of the HAE model (late stage, post-infection for 6 months; middle stage, 3 months; and early stage, 1 month) in C57 mice and explored the negative targeting ability of ICG under the NIR-II-FMI system with an 808 nm laser irradiation for the first time. High-quality NIR-II fluorescence images of HAE microlesions were obtained *in vivo* and *ex vivo* under the NIR-II-FMI system. Meanwhile, normal blood vessels in the liver tissue were clearly observed under the NIR-II-FMI system. This work provides new ideas and tools for a precise diagnosis and shows great potential for the early diagnosis of HAE and intrahepatic diseases.

## 2 Materials and methods

### 2.1 Materials

ICG was purchased from Hangzhou Aoya Biotechnology Co., Ltd. High-glucose Dulbecco’s modified essential medium (DMEM), phosphate-balanced saline (PBS), and fetal bovine serum (FBS) were purchased from Shanghai Aladdin Biochemical Technology Co., Ltd. The Cell Counting Kit-8 (CCK-8) and 4′,6-diamidino-2-phenylindole (DAPI) staining solution were purchased from Biosharp Co., Ltd. RPMI-1640, penicillin, and streptomycin were purchased from Hyclone (Beijing, China). Deionized (DI) water was used in all experimental processes.

### 2.2 Characterization

The morphology of clinical ICG was examined using a transmission electron microscope (TEM, JEM-1230, JEOL, Ltd., Japan) operated in the bright-field mode for morphological evaluation. The amount of ICG was measured with the dynamic light scattering (DLS) method at 25°C using a Zetasizer Nano ZS-90 (Malvern, U.K.). The NIR-II absorption spectrum was obtained from a LAMBDA 750 UV/Vis/NIR spectrophotometer (PerkinElmer). NIR-II fluorescence spectra were measured with an Edinburgh steady/transient fluorescence spectrometer (FLS980). A small animal IVIS Spectrum imaging system (PerkinElmer, United States) was utilized for *ex vivo* and *in vivo* NIR fluorescence imaging (λ_excitation_ = 745 nm; λ_emission_ = 840 nm). A confocal scanning laser microscope (Nikon C2+) was employed for cell fluorescence microscopic imaging. The NIR-II fluorescence spectrum was measured using an Edinburgh steady/transient fluorescence spectrometer. A commercial upright microscope (SOP TOP NIR-II-MS system) was utilized for *in vivo* and *ex vivo* NIR-II fluorescence imaging.

### 2.3 Cells and animals

The human liver cell line L-02 was purchased from the American Type Culture Collection (ATCC, Manassas, VA, United States). The L-02 cells were cultured in DMEM supplemented with 10% FBS and 1% penicillin–streptomycin solution (100 ×) at 37°C in a 5% CO_2_ humidified atmosphere. 6–8-week-old female BALB/c and C57 mice were purchased from the Experimental Animal Center of Xinjiang Medical University. All animal procedures were approved by the Ethics Committee for Animal Experiments of Xinjiang Medical University (approval number: IACUC201902-02). The aforementioned mice were raised in an air-conditioned animal room with a 12 h light/dark cycle and provided with food and water in the Experimental Animal Center of Xinjiang Medical University.

### 2.4 Cytotoxicity

For each CCK-8 assay, L-02 cells were seeded in 96-well plates. The culture medium was replaced with 200 μL of the serum-free culture medium containing different concentrations (30, 60, 90, 120, or 150 μg/mL) of ICG for 24 h. Then, the cells were washed three times with PBS. Next, 90 μL of the fresh medium and 10 μL of the CCK-8 solution were added to each well for 2 h. The optical density (OD) was then measured with a microplate reader at 450 nm.

### 2.5 Confocal laser scanning microscopic cell imaging

L-02 cells (5 × 10^5^ cells per well) were seeded on 12-well plate glass coverslips overnight. Then, the cells were treated with a serum-free medium with 30 μg/mL of ICG for 2 h. After washing them three times with PBS (pH = 7.4), the cells were counterstained with DAPI for 5 min at room temperature. Fluorescent images of cells were acquired using a confocal laser scanning microscope (CLSM). The cellular uptake of ICG and NIR fluorescence images were captured *via* the blue (DAPI, 405 nm excitation) and red (ICG, 640 nm excitation) channels and were further detected using a photomultiplier tube (PMT, detection region: 810–1,000 nm emission).

### 2.6 Establishment of the HAE animal model

*E. multilocularis* protoscoleces (PSCs) were obtained from intraperitoneal lesions from BALB/c mice. Briefly, PSCs were washed five times using the PBS (pH = 7.2, containing 100 μg/mL of penicillin and 100 U/mL of streptomycin). After being counted under a microscope (DMI4000B, Leica, Germany), about 2,000 of *E. multilocularis* PSCs were resuspended in 1 mL of PBS and were injected into the intraperitoneal cavity of 8–10-week-old BALB/c mice. The vitality was determined by staining PSCs with 0.1% of methylene blue, and a viability of over 95% was utilized. The experimental mice were inoculated *via* the hepatic portal vein with different doses (1,000–2,000) of PSCs in saline, while the control mice were injected with the PBS × 3. The experimental mice were sacrificed at 4 (early stage), 12 (middle stage), and 24 (late stage) weeks after injection. The livers were sectioned and carefully screened for hepatic lesions for a preliminary assessment (H&E analysis) of the *E. multilocularis* infection at these time points. For the identification of neovascularization in HAE-infected livers, anti-mouse-CD34 antibodies were used to probe the liver sections according to the previously published methods (Wang et al., 2016).

### 2.7 NIR-I fluorescence imaging

ICG dissolved in various solutions was used as a dye. The images of HAE-infected mice and livers were obtained using an IVIS spectrum imaging system. First, ICG in different solutions (DI water, PBS, DMEM, and FBS) at different concentrations (0.001 mg/mL, 0.01 mg/mL, and 0.1 mg/mL) in tubes was imaged under this NIR fluorescence imaging system (745 nm laser excitation and 840 nm emission). Then, these ICG solutions were intramuscularly injected into mice that were then imaged using our NIR-I fluorescence imaging system. 24 h after injection, these mice were sacrificed, and their livers were collected for *in vitro* NIR fluorescence imaging.

### 2.8 Establishment of the NIR-II fluorescence microscopic imaging system

The NIR-II-FMI system includes a fluorescence microscope (SOP TOP NIR-II-MS system) equipped with an 808 nm laser as an excitation source and an InGaAs camera (640 × 512 pixels; detection wavelength: 900–1700 nm). The laser beam comes out from the excitation source, then passes through a 980 nm long-pass dichroic mirror, and a ×3.3 infrared air objective lens (LSM04; working distance = 42.3 mm). The fluorescence signal intensity excited in the targeted tissues or organs was then reflected along the original light path. NIR-II fluorescence images were collected and recorded with the InGaAs camera after passing through the long-pass dichroic mirror and a 900/1,000 nm long-pass filter.

### 2.9 NIR-II fluorescence microscopic imaging of L-02 cells

The L-02 cells (2.5 × 10^6^ cells per well) were seeded on 12-well plate glass coverslips overnight. Then, the cells were treated with a serum-free medium with 30 μg/mL of ICG for 2 h. After washing them three times with the PBS (pH = 7.4), the fluorescent images of the L-02 cells were acquired using the NIR-II-FMI system under an 808 nm laser irradiation.

### 2.10 NIR-II fluorescence microscopic imaging of *E. multilocularis* metacestode

PSCs from *E. multilocularis* were collected from the peritoneal cavity of Mongolian jirds. The sedimented PSCs were digested with 1% (w/v) pepsin (Sigma-Aldrich, Louis, MO, United States) at pH = 2.0 (adjusted with 2 M HCl) in Hank’s buffer for 20 min at 37°C. After five washes with the PBS, the viability of PSCs was determined by staining with 0.1% methylene blue, with dead PSCs staining blue. PSCs with ≥ 95% viability were subsequently used for cultivation and infection. The culture used the same method as described previously (Zhang et al., 2017). The cultured microcysts were seeded in 12-well culture plates at 37°C in the presence of 5% of CO_2_. Then, the cysts were treated with the serum-free medium with 30 μg/mL of ICG for 1.5 h. After washing them three times with the PBS (pH = 7.4), NIR-II fluorescence images of these cysts were acquired using the NIR-II-FMI system under an 808 nm laser irradiation.

### 2.11 *In vivo* NIR-II fluorescence microscopic imaging

Each mouse’s abdomen was shaved before injecting it with a dye solution. The mice injected with 200 μL of ICG (0.1 mg/mL) were used as an experimental group versus the control mice injected with the same amount of 1 × PBS. Then, the two groups of mice were anesthetized and imaged by the NIR-II-FMI system. Next, the liver tissue of these mice was taken out and imaged under the same system.

### 2.12 Tissue sampling and histopathological analysis

For liver histopathology, all liver tissues were taken from the mice and fixed in 10% buffered formalin and then embedded in paraffin. The paraffin-embedded liver tissue was sectioned at a thickness of 4 μm and mounted on glass slides and then stained with hematoxylin–eosin (H&E). For the liver fibrosis Masson analysis, the sections were stained using the picric acid-Sirius red staining technique to evaluate the collagen fibers, as described previously. This was assessed at ×100 magnifications as total sections.

### 2.13 *In vivo* CT imaging

*In vivo* CT images of HAE-infected mice were obtained using a CT scanning instrument (Philips, Brilliance Big Bore). The infected mice were anesthetized and placed on the CT scanning system in the regular prone position (fixed to a foam board), with the mice placed in the middle of the bed, aligned along the median sagittal plane. The mice were then imaged from the head to the root of their tail using the 16 slice CT spiral scanning mode with a scanning thickness of 0.8 mm.

### 2.14 Statistical analysis

The all data are shown as mean ± SD. Every experiment was conducted for three replicates (*n* = 3). For statistical analysis, a Mann−Whitney U test was performed using Origin 8 software. ImageJ software was used to analyze the fluorescence images. The values of *p* < 0.05 were considered significant.

## 3 Results

### 3.1 Characterization of the ICG solution

**FIGURE 1:**
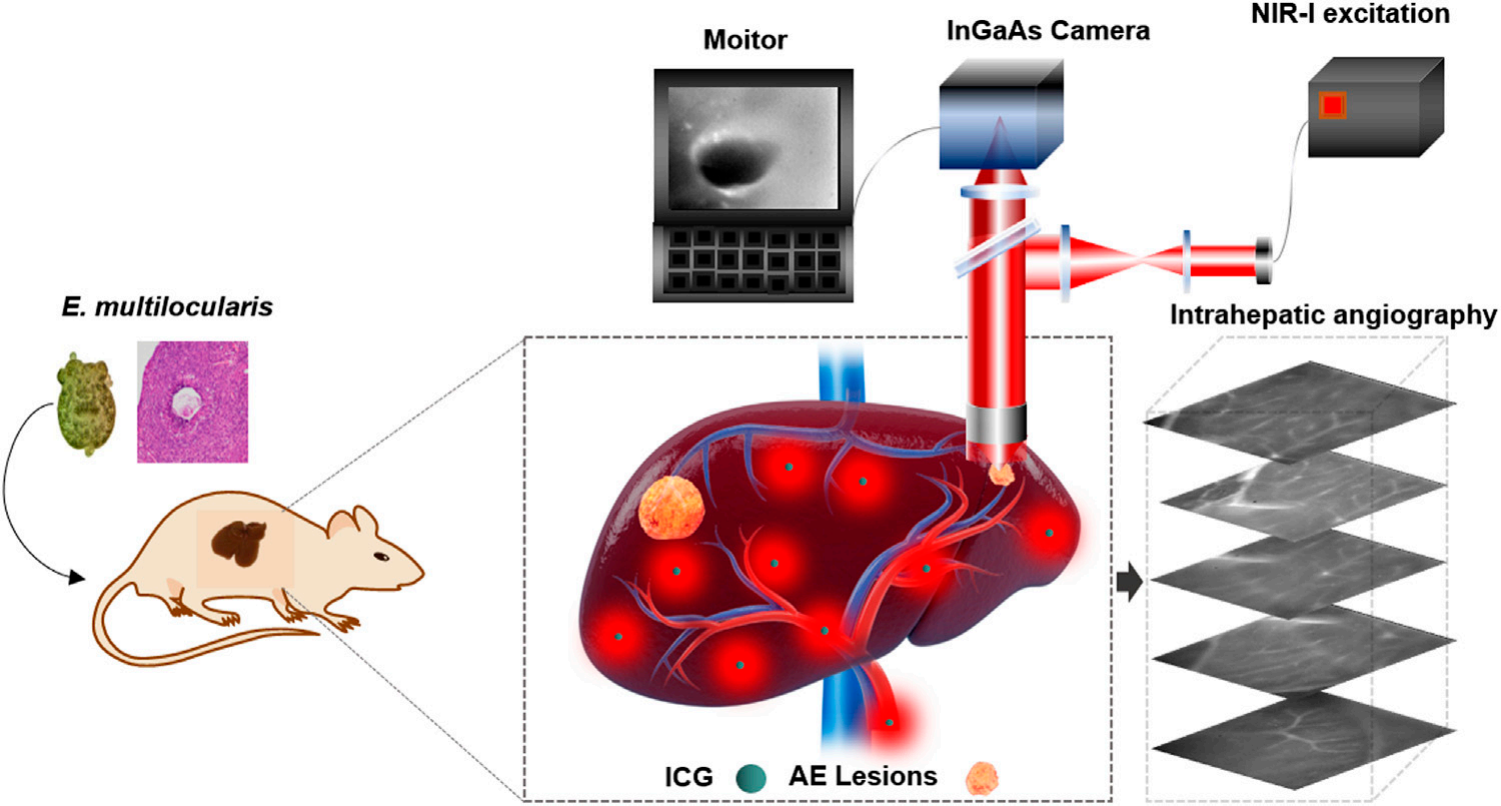
Schematic illustration of NIR-II fluorescence imaging for recognition of hepatic microlesions in alveolar echinococcosis and intrahepatic angiography.

**FIGURE 2:**
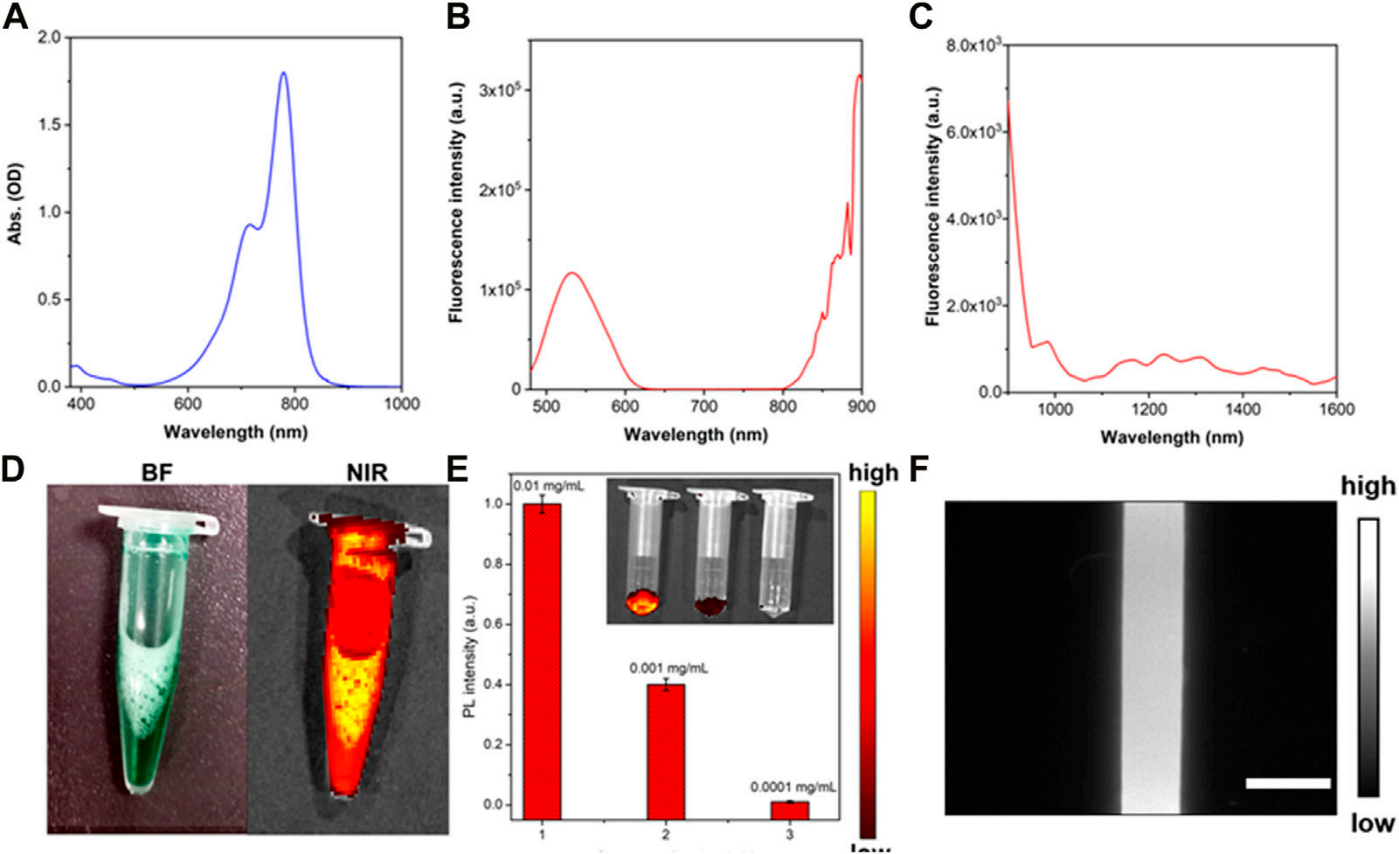
Characterization of clinical ICG. **(A)** Absorption spectrum, **(B)** NIR-I fluorescence spectrum (λ_excitation_ = 450 nm), and **(C)** NIR-II fluorescence spectrum of ICG in the FBS (λ_excitation_ = 645 nm). **(D)** NIR-I fluorescence images of ICG in the FBS, **(E)** NIR-I fluorescence intensity of ICG in the FBS at different concentrations (0.01 mg/mL, 0.001 mg/mL, and 0.0001 mg/mL). λ_excitation_ = 745 nm and λ_emission_ = 840 nm. Inset: NIR-I fluorescence images of ICG (in FBS) with different concentrations of 0.01 mg/mL, 0.001 mg/mL, and 0.0001 mg/mL. **(F)** NIR-II fluorescence microscopic images of ICG in the FBS under an 808 nm laser irradiation.

Clinical ICG was utilized for negative NIR-II fluorescence microscopic imaging of the *in vivo* and *in vitro* HAE model (Figure 1). First, the optical property of clinical ICG was studied in detail. Transmission electron microscopy and dynamic light scattering were performed to analyze the size and morphology of ICG. As shown in Supplementary Figure S1, ICG in water showed an average size of about 260.4 nm. As shown in Figure 2, ICG showed strong NIR-I fluorescence in the FBS.31. Thus, the absorption spectrum of ICG in the FBS was measured as shown in Figure 2A, at a peak wavelength of 780 nm. Fluorescence spectra of ICG in the FBS were further recorded in the NIR-I (Figure 2B) and NIR-II (Figure 2C) spectral wavelength regions. Most of the fluorescence spectra were in the NIR-II fluorescence spectral region. Additional NIR-I fluorescence images for ICG in the FBS were obtained by *in vivo* small animal NIR fluorescence imaging. A strong NIR-I fluorescence intensity was detected even at a low concentrations of ICG in the FBS (0.01 mg/mL), as shown in Figures 2D,E. Then, a capillary glass tube containing 0.01 mg/mL of ICG (in FBS) was imaged under the NIR-II-FMI system. As shown in Figure 2F, strong NIR-II fluorescence signals were observed from ICG under an 808 nm laser irradiation.

### 3.2 Cell uptake

**FIGURE 3:**
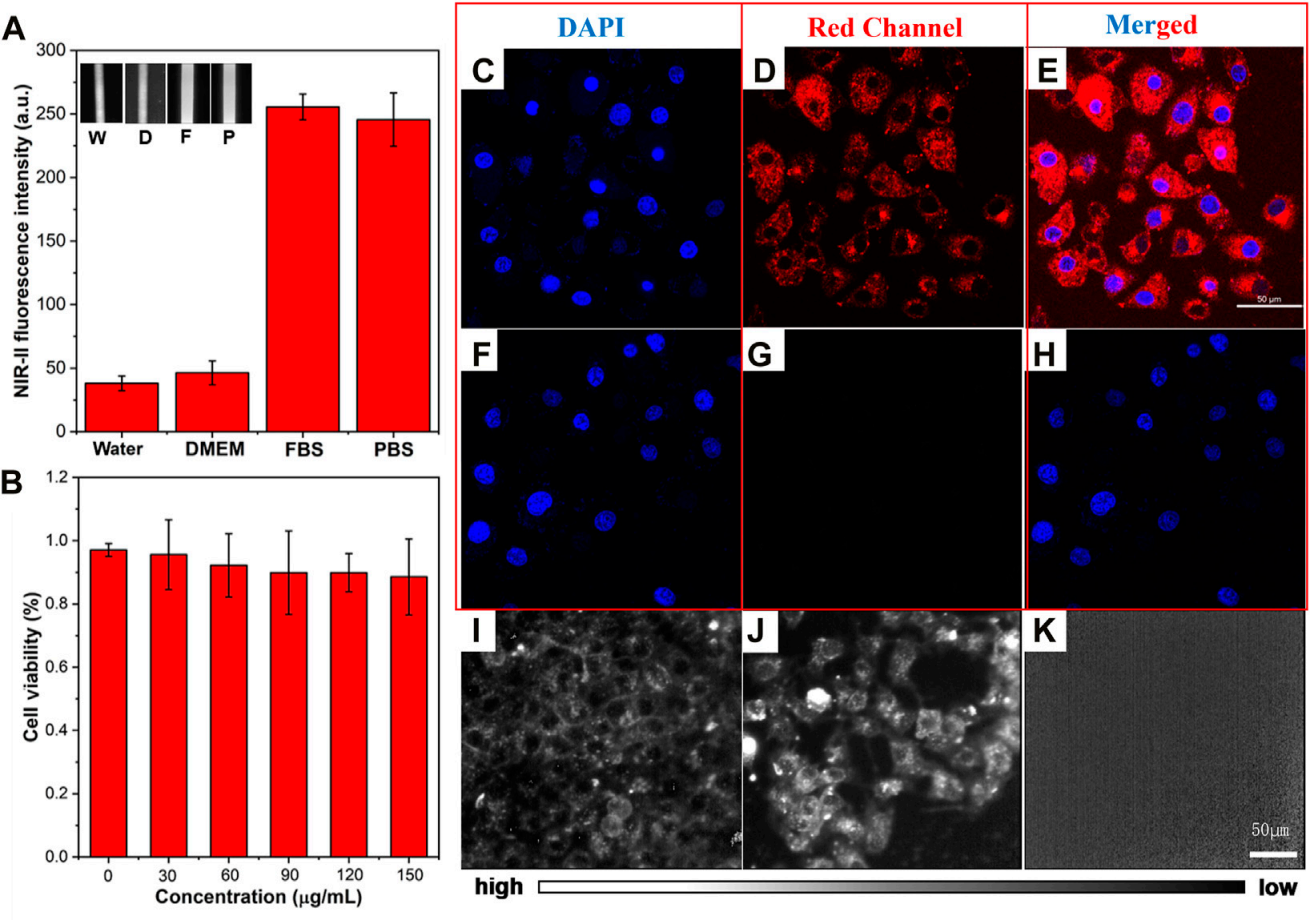
**(A)** NIR-II fluorescence intensity of ICG in different solutions. Inset: NIR-II fluorescence microscopic images of ICG in water (W), DMEM **(D)**, FBS **(F)**, and PBS (P) at the same concentration of 0.1 mg/mL (λ_excitation_ = 808 nm). **(B)** Cell viability of ICG in L-02 liver cells. **(C–E)** CLSM images of L-02 liver cells incubated with ICG and **(F–H)** FBS, under a 640 nm laser excitation and 800–1,000 nm emission. **(I–J)** NIR-II fluorescence microscopic images of L-02 liver cells incubated with ICG and **(K)** control cells under an 808 nm laser irradiation (scale bar = 50 μm).

The NIR-II fluorescence intensity of ICG (0.01 mg/mL) in different solutions (water, DMEM, FBS, and PBS) was analyzed using the NIR-II-FMI system. As shown in Figure 3A, ICG in the FBS solution showed the strongest NIR-II fluorescence signals compared with the other three solutions. This may be due to the different solubility and fluorescence quantum yield of ICG in water, DMEM, PBS, and FBS (Yu et al., 2019). Furthermore, the cell viability of ICG in the liver cell line L-02 was analyzed at different concentrations (0 μg/mL, 30 μg/mL, 60 μg/mL, 90 μg/mL, 120 μg/mL, and 150 μg/mL). As shown in Figure 3B, even at a high concentration, the low toxicity of ICG was confirmed in the normal liver cells L-02. Then, confocal laser scanning microscopic (CLSM) images of ICG incubated liver cells L-02 were collected. As shown in Figures 3C–E, in the experimental group, strong fluorescence signals from the DAPI-labeled nucleus of liver cells were detected in the blue channel (under a 405 nm laser excitation). Meanwhile, strong fluorescence signals were observed from the cytoplasm of ICG-stained liver cells in the red channel (under a 640 nm laser excitation). In the control group (without the treatment of ICG), as shown in Figures 3F–H, fluorescence signals from the DAPI-labeled nucleus could be detected. Furthermore, the ICG incubated liver cell line L-02 was imaged by the NIR-II-FMI system under an 808 nm laser irradiation. As shown in Figures 3I,J, strong NIR-II fluorescence signals from the ICG-labeled cytoplasm could be observed, while in the control group without the treatment of ICG (Figure 3K), no fluorescence signals could be detected.

### 3.3 *In/ex vivo* NIR fluorescence imaging

**FIGURE 4:**
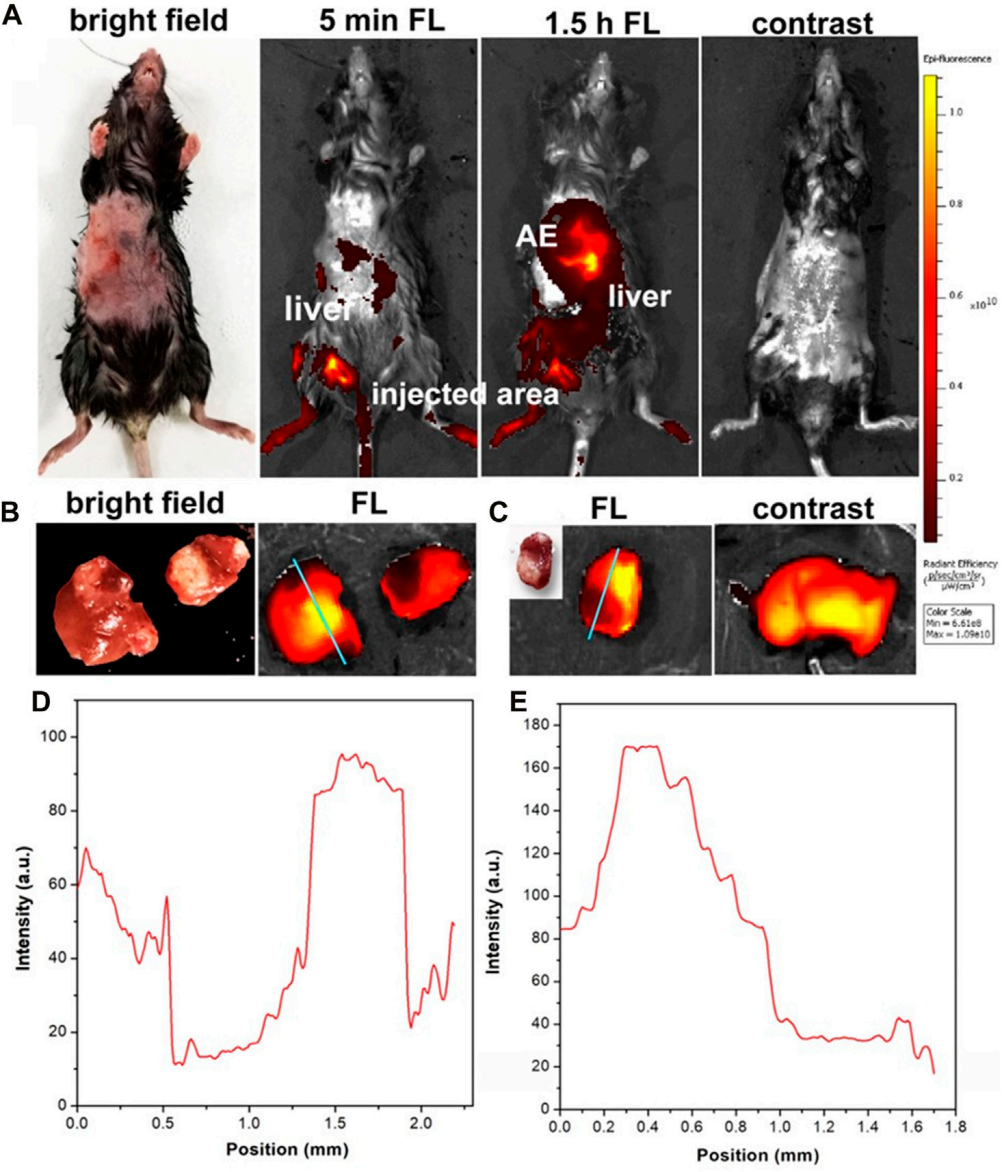
**(A)** *In vivo* NIR fluorescence images of HAE-infected mice at the late stage with and without the injection of ICG. **(B, C)** were the liver tissue from the late stage of the HAE-infected mouse after injection of ICG and in the normal mouse (control). **(D, E)** were the FWHM analysis of **(B, C)**. λ_excitation_ = 740 nm and λ_emission_ = 840 nm.

C57 mice (*n* = 60, female, and aged 6–8 weeks) were utilized for the establishment of the HAE mouse model at the late stage. Then, 200 μL of ICG (concentration: 0.01 mg/mL in FBS) was intramuscularly injected into these HAE mice after being anesthetized. The abdomen of each mouse was shaved and imaged using an IVIS fluorescence imaging system. Strong NIR-I fluorescence signals were observed from the normal liver of these mice, and the HAE lesion displayed as a black area (negative label), with most microlesions appearing as a round shape (Figure 4A). These HAE-infected livers were then taken out from these mice and imaged under the same system using the same conditions.

NIR-I fluorescence signals were captured from the normal area of the liver tissue, while there were no fluorescence signals (negative fluorescence image) detected from the HAE lesion area, as shown in Figures 4B,C. In the control group, normal mice without infection were injected with the PBS containing the same amount of ICG. Strong NIR-I fluorescence signals were observed and measured. Then, a full width half maximum (FWHM) analysis was conducted for these two tissue sections and high contrast (signal-to-noise ratio, SBR) images were obtained as shown in Figure 4D (SBR = 6.6; FWHM = 0.48 mm) and Figure 4E (SBR = 8; FWHM = 0.6 mm). Hence, the *in vivo* and *in vitro* NIR-I fluorescence imaging results illustrated the negative targeting ability of ICG in the late-stage HAE mice.

### 3.4 *In vivo* and *ex vivo* NIR-II microscopic fluorescence imaging of HAE lesions

**FIGURE 5:**
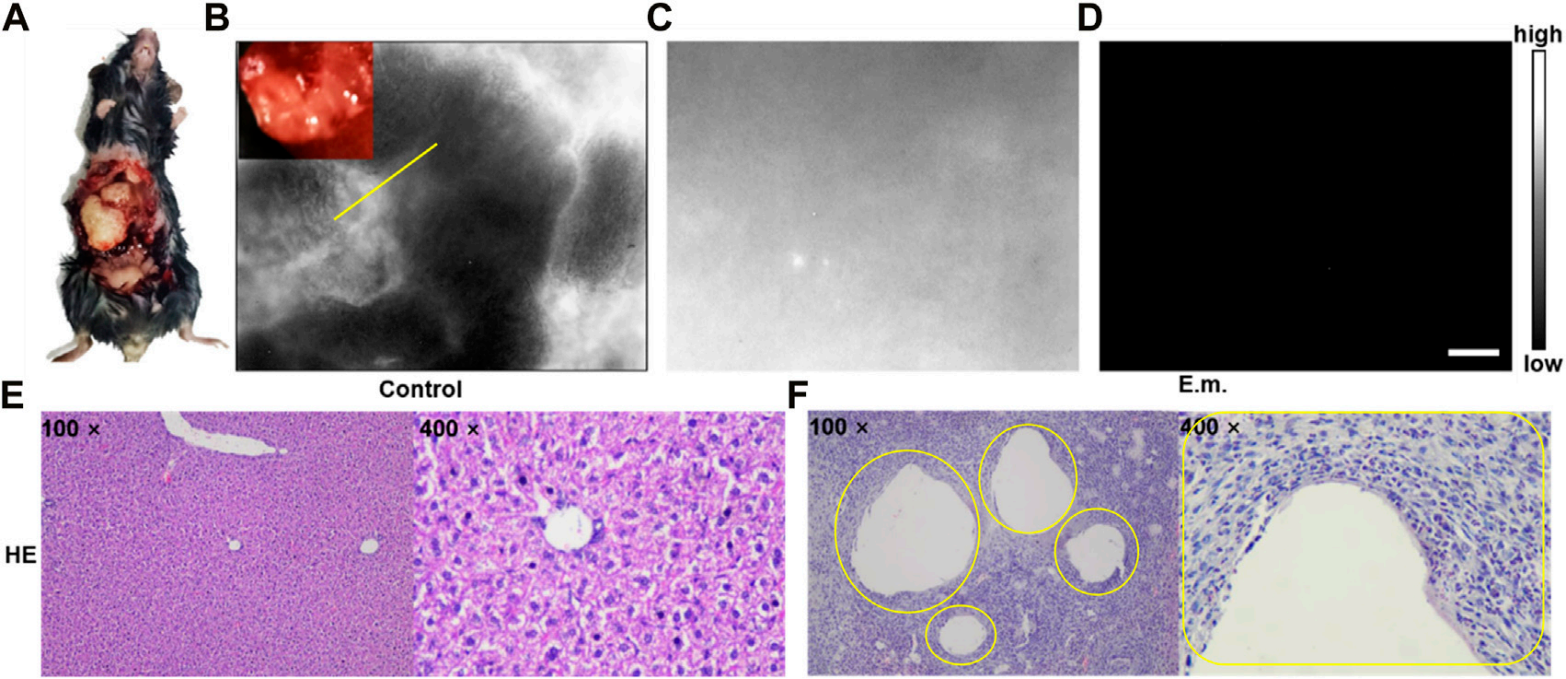
**(A–B)** NIR-II fluorescence microscopic images of liver tissues at the late stage of HAE mice with the injection of ICG. **(C)** NIR-II fluorescence microscopic images of normal liver tissues injected with ICG and **(D)** PBS under an 808 nm laser irradiation (scale bar = 50 μm). Corresponding microscopic images of **(E)** H&E stains on the normal liver tissue as the control group and **(F)** liver tissues taken from the HAE mice as the experimental group (inset: the lesion location is shown by the yellow circle).

In order to obtain more detailed information from liver and microlesions, NIR-II fluorescence microscopic imaging was conducted on the HAE mice at the late-stage post-injection of ICG. As shown in Figure 5A, the *E. multilocularis*-infected livers were then taken and imaged using the NIR-II-FMI system under an 808 nm laser irradiation (Supplementary Figure S2). As shown in Figure 5B, the fine structure around the HAE lesion can be observed with a high signal-to-noise ratio and good contrast (SBR = 3.16; FWHM = 0.35 mm).

No notable negative targeted areas could be observed in the liver of normal mice (Figure 5C), and no NIR-II fluorescence signals could be detected in the control group of the *E. multilocularis*-infected mouse post-injection of the PBS (Figure 5D) under the same NIR-II microscopic fluorescence imaging condition. In addition, as shown in Figures 5E,F, hematoxylin–eosin-stained normal liver tissue as the control group and the liver tissue taken from the HAE mice as the experimental group yielded the same results with Figures 5B,C.

**FIGURE 6:**
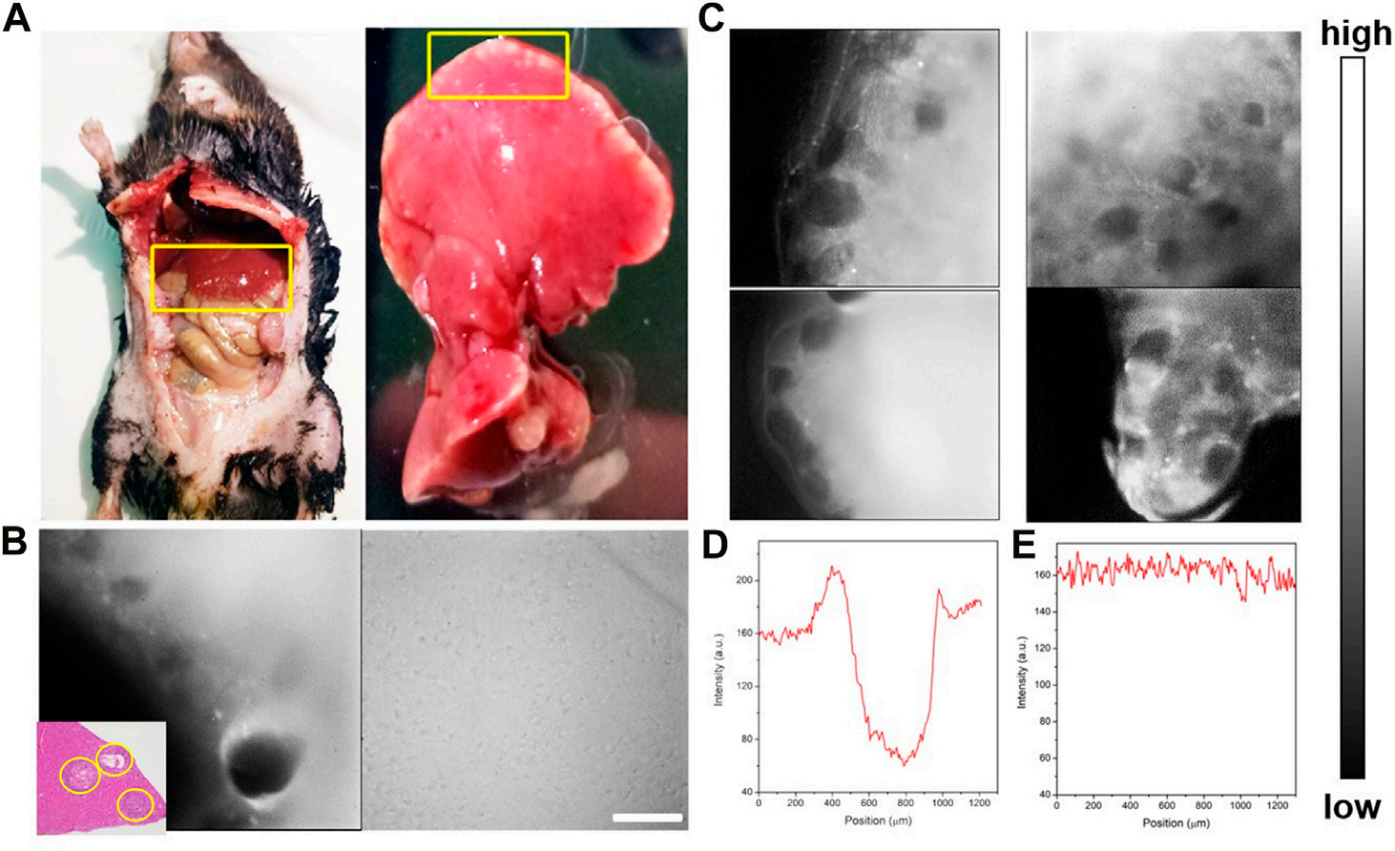
**(A)** Bright-field imaging of the C57 mouse and its liver tissue infected with HAE. **(B)** NIR-II fluorescence microscopic images of liver tissue at the middle stage of HAE infection post ICGinjection. **(C)** NIR-II fluorescence microscopic images of the HAE-infected liver (left) and the normal liver tissue injected with ICG (right) under an 808 nm laser irradiation (inset: H&E-stained liver tissues taken from HAE mice; the lesion location is shown by the yellow circle). **(D, E)** Corresponding FWHM analysis of **(C)** indicated by yellow.

Furthermore, a middle-stage *E. multilocularis*-infected mouse model was established. Micro-AE lesions at the edge of liver tissues were visible to the naked eye (Figure 6A). Then, the *E. multilocularis*-infected liver was imaged by the NIR-II-FMI system using the same conditions. As shown in Figure 6B, many black holes, which were HAE microlesions could be clearly seen *via* a ×3.3 objective more than the naked eyes could see. The FWHM analysis further demonstrated the high contrast and clear identification ability of NIR-II microscopic fluorescence imaging. As shown in Figure 6C, the high spatial resolution image was further analyzed with a high SBR of 7.2 mm and FWHM of 0.4 mm (Figures 6D,E).

In order to verify the negative targeting ability of ICG-assisted NIR-II fluorescence microscopic imaging for the recognition of microlesions, an *E. multilocularis*-infected mouse model of the early stage was established in 1 month post-infection of BALB/c mice.

**FIGURE 7:**
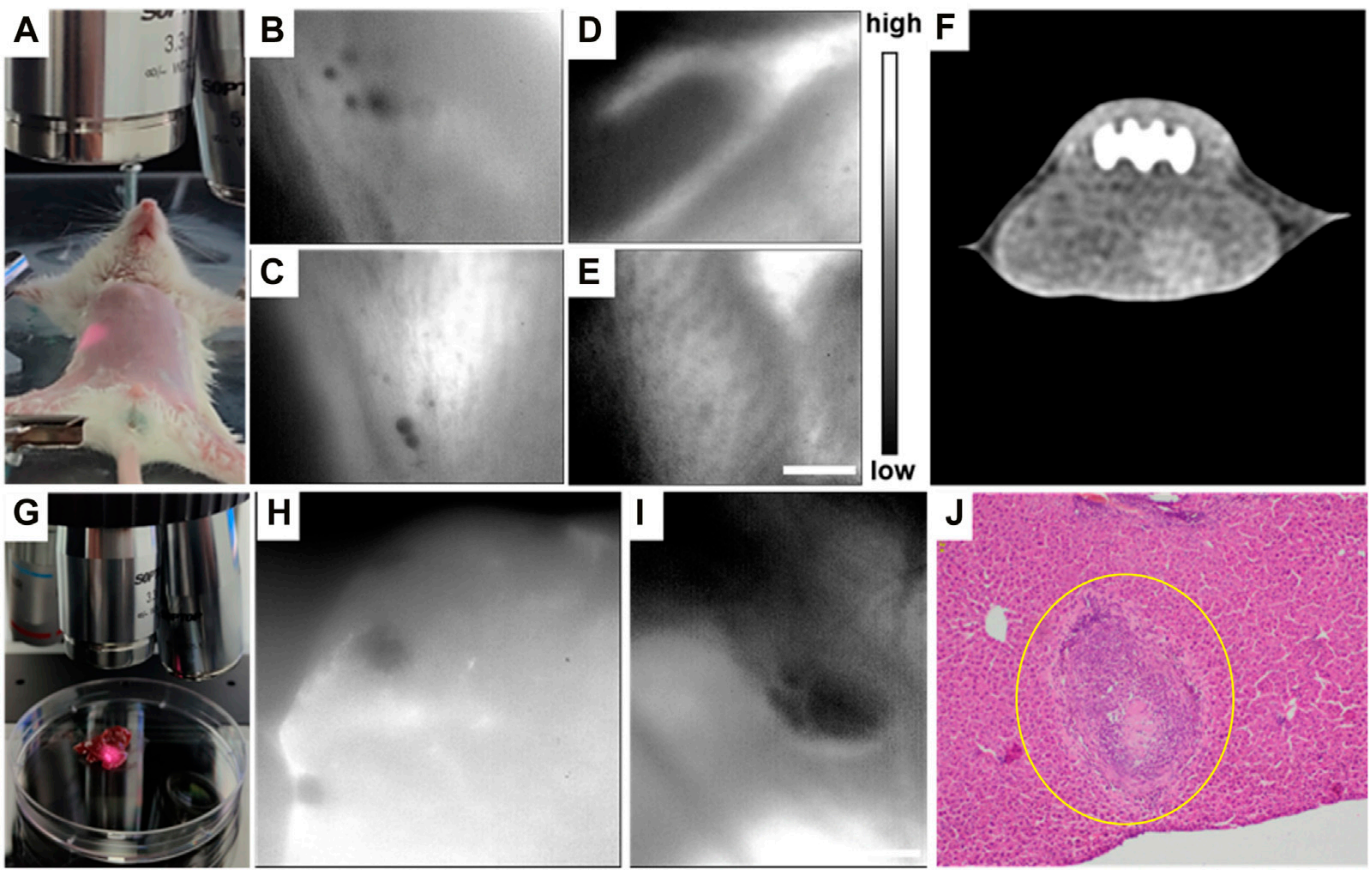
**(A)** Typical NIR-II fluorescence microscopic image of **(E)** multilocularis-infected mice at the early stage. **(B, C)** I*n vivo* NIR-II fluorescence microscopic images of **(E)** multilocularis-infected mice post ICG injection. **(D, E)** NIR-II fluorescence microscopic images of portal vein angiography injected with ICG. **(F)** Typical CT image of **(A)**. **(G)** Typical image of **(E)** multilocularis-infected liver tissues at the early stage under the NIR-II-FMI system. **(H–I)** *In vitro* NIR-II fluorescence microscopic images of **(E)** multilocularis-infected liver tissues and the corresponding H&E images. λ_excitation_ = 808 nm. **(J)** H&E-stained liver tissues taken from HAE mice (inset: the lesion location is shown by the yellow circle).

An *E. multilocularis*-infected living mouse was still placed in the aforementioned NIR-II-FMI system under an 808 nm laser irradiation and imaged *via* a ×3.3 objective, as shown in Figure 7A. The infected lesion showed notable negative targeting areas (Figures 7B,C). Meanwhile, the blood vessels under the skin could be clearly observed *in vivo* (Figures 7D,E), while the lesion area of the same mouse could not be detected by CT imaging (Figure 7F). These results show that the NIR-II-FMI system possess superior spatial resolution and high contrast for early-stage microlesions. Additionally, the infected liver tissue was removed and imaged under the same conditions (Figure 7G). Microlesions could be imaged clearly (Figures 7H,I). The same area of the liver tissue was further cut out and H&E staining analysis was subsequently performed. As shown in Figure 7J, H&E staining of the liver tissue from the HAE mice further confirmed the lesion area.

Then, the *E. multilocularis*-infected lesions could be observed clearly *via* the pathological sections. Additionally, microcysts were incubated in the DMEM and stained with the ICG aqueous solution. After washing them three times with the PBS, the cysts were imaged *via* the NIR-II NIR-II-FMI system under an 808 nm laser irradiation.

**FIGURE 8:**
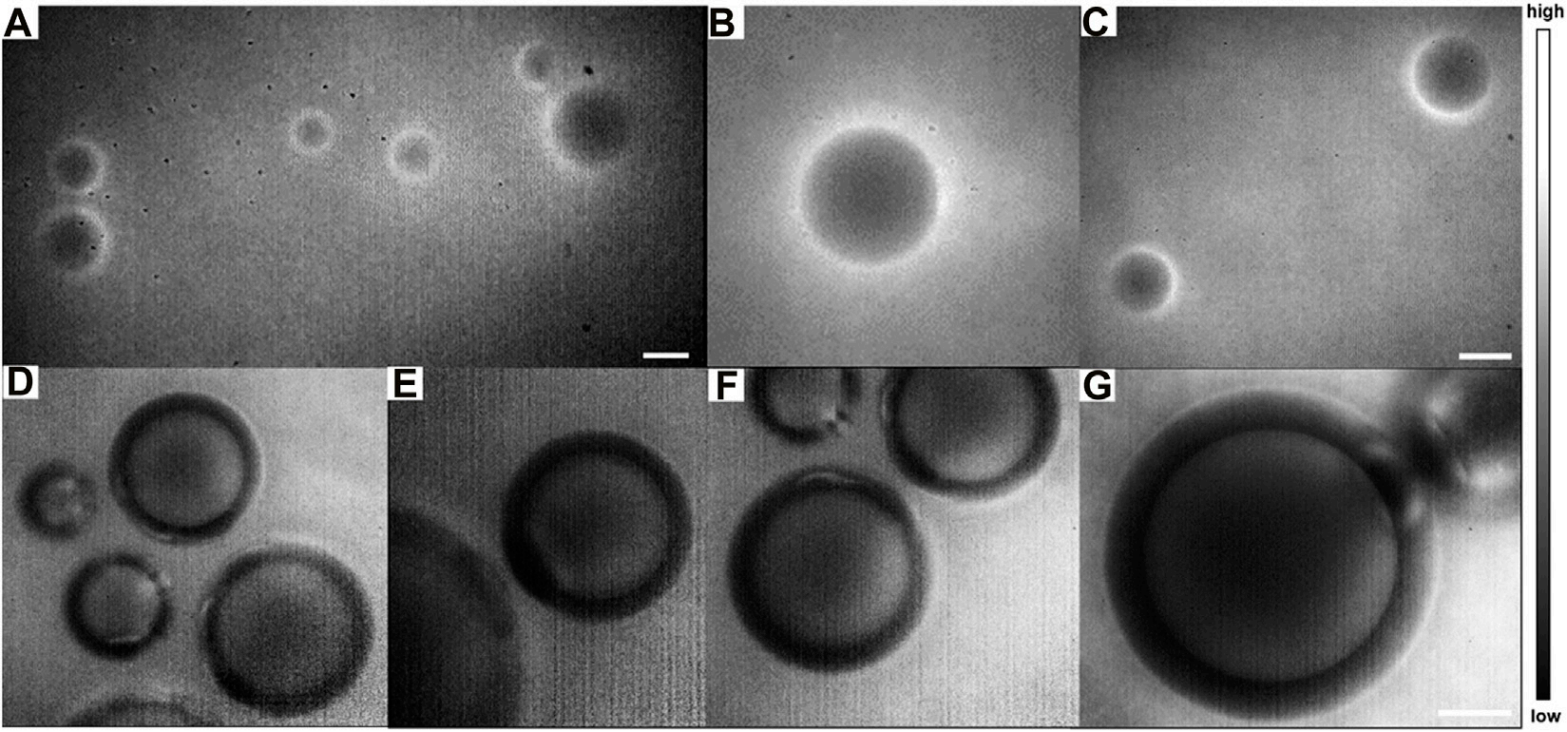
NIR-II fluorescence microscopic images of ICG-stained *E. multilocularis* vesicles obtained from cultured protoscoleces *in vitro* on day 60 **(A)** in a large field of view under a ×10 objective and **(B–C)** by local magnification. **(D–E)** Magnification fields of *E. multilocularis* vesicles post-treatment with ICG under a **(F)** ×25 objective and **(G)** ×50 objective (scale bars: 100 μm) under an 808 nm laser irradiation.

As shown in Figures 8A–G, the submicron microstructure is visible with a high spatial resolution and contrast. The inside of the lesions with *E. multilocularis* metacestode was black and no fluorescent signal was detectable. This result further validates our previous animal experimental conclusions, which was the negative targeting ability of ICG for HAE. As far as we know, this was the first time that the NIR-II-FMI system was utilized with the assistance of ICG as a negative targeting fluorescent probe for HAE diseases *in vitro* and *in vivo.*

**FIGURE 9:**
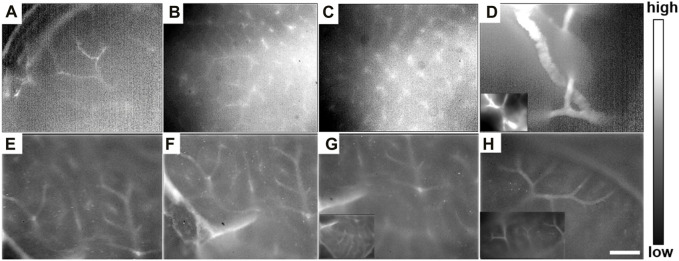
**(A–H)** *In vitro* NIR-II fluorescence microscopic images of intrahepatic angiography post-injection of ICG under ×25 objective, with an 808 nm laser irradiation.

Meanwhile, after the liver tissue was taken from the body of the mouse and imaged under the NIR-II-FMI system post-injection of ICG, abundant tiny blood vessels in the liver could be clearly identified. The distribution of blood vessels in different regions of the normal liver could be detected (Figures 9A–H). The rich capillary structure in liver tissues, large blood vessels, and portal vein vessels were observable at a high resolution and high signal-to-noise ratio. As far as we know, this is the first time the NIR-II-FMI system was used to realize the visualization of blood vessel distribution in the liver with the assistance of ICG as a fluorescent probe. Our results provide technical support for liver vascular diseases and provide a very good basis for future clinical transformations.

## 4 Discussion

It is essential to utilize advanced imaging modalities to achieve highly efficient and precise diagnoses in biomedicine. Especially for the early diagnosis of diseases, accurate imaging of structures is of great importance. HAE is a cancer-like disease that has caused great damage to humans. However, until now, there has been no effective method for the efficient early diagnosis of HAE microlesions.

NIR-II fluorescence imaging with advantages of minimal light scattering and negligible tissue autofluorescence, exhibits its superiority for deep-tissue imaging with a high spatial resolution. The combination of NIR-II fluorescence imaging with microscopic fluorescence imaging has promoted a novel kind of NIR-II-FMI system. Compared with a simple living imaging system, NIR-II-FMI obtains high-quality microscopic images of tiny structures and small lesions *in vivo*.

In addition, the fluorescence probe with good NIR-II fluorescence emission and biosafety and targeting ability is also critical for highly efficient NIR-II fluorescence imaging. Among the now-existing NIR-II fluorescence probes, ICG is a favorable one because of its excellent properties of bioimaging. Moreover, ICG has good *in vivo* and *ex vivo* labeling ability. Thus, the ICG-assisted NIR-II-FMI system under an 808 nm laser excitation shows great potential in the detection of small lesions.

In this work, the fluorescence properties of ICG were measured and analyzed. ICG exhibited strong NIR-II fluorescence signals under the NIR-II-FMI system with an 808 nm laser irradiation. Moreover, ICG showed an excellent labeling ability for the normal liver tissue and cells under the NIR-II-FMI system. As for the HAE-infected mouse model, ICG showed negative targeting ability for *in vivo* and *ex vivo* conditions. The NIR-II fluorescence images of *E. multilocularis* vesicles and *E. multilocularis*-infected livers at different stages had illustrated that ICG could not enter the lesions, which could delineate the lesions in a reverse targeting manner. However, for the normal liver tissue, the blood vessels in the liver tissue could be displayed. Thus, according to these exciting results, the developed NIR-II-FMI system assisted with ICG has great potential in future clinical applications of the early diagnosis of liver diseases.

## 5 Conclusion

In this work, the NIR-II-FMI system with assistance from ICG as a negative targeting fluorescent probe was successfully applied for the first time for detecting HAE microlesions at the different infection stages of *E. multilocularis*, such as the early (1 month), middle (3 months), and late stage (6 months) in the mouse model infected with *E. multilocularis*. The *in*/*ex vivo* HAE microlesions that could not be recognized by CT could be observed clearly by NIR-II fluorescence microscopic imaging. Clear intrahepatic angiography was also obtained *via* the same imaging methods and conditions under the NIR-II-FMI system with the assistance of ICG. This method has great value for the early diagnosis and prognosis evaluation of HAE and the related intrahepatic diseases.

## Data Availability

The raw data supporting the conclusions of this article will be made available by the authors, without undue reservation.

## References

[B1] Abuseir S. Nagel-Kohl U. Wolken S. Strube C. (2013). An immunoblot for detection of Taenia saginata cysticercosis. Parasitol. Res. 112 (5), 2069–2073. 10.1007/s00436-013-3368-5 23483261

[B2] Alifu N. Zebibula A. Qi J. Zhang H. Sun C. Yu X. (2018). Single-Molecular near-infrared-II theranostic systems: Ultrastable aggregation-induced emission nanoparticles for long-term tracing and efficient photothermal therapy. ACS Nano 12 (11), 11282–11293. 10.1021/acsnano.8b05937 30345739

[B3] Bhutani N. Kajal P. (2018). Hepatic echinococcosis: A review. Ann. Med. Surg. (Lond) 36, 99–105. 10.1016/j.amsu.2018.10.032 30450204 PMC6226561

[B4] Carr J. A. Franke D. Caram J. R. Perkinson C. F. Saif M. Askoxylakis V. (2018). Shortwave infrared fluorescence imaging with the clinically approved near-infrared dye indocyanine green. Proc. Natl. Acad. Sci. U. S. A. 115 (17), 4465–4470. 10.1073/pnas.1718917115 29626132 PMC5924901

[B5] Chen S. Kong J. J. Qiu Y. W. Zhang S. Qin Y. Wang W. (2019). *Ex vivo* liver resection and autotransplantation versus allotransplantation for end-stage hepatic alveolar echinococcosis. Int. J. Infect. Dis. 79, 87–93. 10.1016/j.ijid.2018.11.016 30496849

[B6] Chen Z. Y. Wang Y. X. Yang F. Lin Y. Zhou Q. L. Liao Y. Y. (2014). New researches and application progress of commonly used optical molecular imaging technology. Biomed. Res. Int. 2014, 1–22. 10.1155/2014/429198 PMC3947735 24696850

[B7] Copeland N. K. Aronson N. E. (2015). Leishmaniasis: Treatment updates and clinical practice guidelines review. Curr. Opin. Infect. Dis. 28 (5), 426–437. 10.1097/QCO.0000000000000194 26312442

[B8] Deplazes P. Rinaldi L. Rojas C. A. A. Torgerson P. R. Harandi M. Romig T. (2017). Global distribution of alveolar and cystic echinococcosis. Adv. Parasitol. 95, 315–493. 10.1016/bs.apar.2016.11.001 28131365

[B9] Dezsényi B. Strausz T. Makrai Z. Csomor J. Danka J. Kern P. (2017). Autochthonous human alveolar echinococcosis in a Hungarian patient. Infection 45 (1), 107–110. 10.1007/s15010-016-0918-7 27352256

[B10] Fan Y. Zhang F. Zhou H. Ding B. Li A. Lin J. (2019). Quaternary ammonium salt based NIR-II probes for *in vivo* imaging. Adv. Opt. Mater 7 (15), 1900229. 10.1002/adom.201900229 32983835 PMC7517706

[B11] Feng Z. Yang Y. Zhang J. Wang K. Li Y. Xu H. (2019). *In vivo* and *in situ* real-time fluorescence imaging of peripheral nerves in the NIR-II window. Nano Res. 12, 3059–3068. 10.1007/s12274-019-2552

[B12] Giorgi C. Bonora M. Sorrentino G. Missiroli S. Poletti F. Suski J. M. (2015). P53 at the endoplasmic reticulum regulates apoptosis in a Ca2+-dependent manner. Proc. Natl. Acad. Sci. U. S. A. 112 (6), 1779–1784. 10.1073/pnas.1410723112 25624484 PMC4330769

[B13] Gottstein B. Stojkovic M. Vuitton D. A. Millon L. Marcinkute A. Deplazes P. (2015). Threat of alveolar echinococcosis to public health-a challenge for Europe. Trends Parasitol. 31 (9), 407–412. 10.1016/j.pt.2015.06.001 26115902

[B14] Hemphill A. Stadelmann B. Rufener R. Spiliotis M. Boubaker G. Muller J. (2014). Treatment of echinococcosis: Albendazole and mebendazole-what else? Parasite 21, 70. 10.1051/parasite/2014073 25526545 PMC4271654

[B15] Hong G. Antaris A. L. Dai H. (2017). Near-infrared fluorophores for biomedical imaging. Nat. Biomed. Eng. 1, e0010. 10.1038/s41551-016-0010

[B16] Li D. Gao Q. Liu J. Feng Y. Ning W. Dong Y. (2015). Knowledge, attitude, and practices (KAP) and risk factors analysis related to cystic echinococcosis among residents in Tibetan communities, Xiahe County, Gansu Province, China. China. Acta Trop. 147, 17–22. 10.1016/j.actatropica.2015.02.018 25757370 PMC4441730

[B17] Li Y. P. Ma Z. G. Tuxun T. Li Z. D. Meng Y. Chen X. (2020). The application of laparoscopy combined with indocyanine green fluorescence imaging technique for hepatic cystic echinococcosis. BMC Surg. 20 (1), 249. 10.1186/s12893-020-00911-8 33092557 PMC7579955

[B18] Lin J. Zeng X. Xiao Y. Tang L. Nong J. Liu Y. (2018). Novel near-infrared II aggregation-induced emission dots for *in vivo* bioimaging. Chem. Sci. 10 (4), 1219–1226. 10.1039/c8sc04363a 30774922 PMC6349025

[B19] Mcmanus D. P. Gray D. J. Zhang W. Yang Y. (2012). Diagnosis, treatment, and management of echinococcosis. BMJ 344, e3866. 10.1136/bmj.e3866 22689886

[B20] Mihmanli M. Idiz U. O. Kaya C. Demir U. Bostanci O. Omeroglu S. (2016). Current status of diagnosis and treatment of hepatic echinococcosis. World J. Hepatol. 8 (28), 1169–1181. 10.4254/wjh.v8.i28.1169 27729953 PMC5055586

[B21] Nicolao M. C. Elissondo M. C. Denegri G. M. Goya A. B. Cumino A. C. (2014). *In vitro* and *in vivo* effects of tamoxifen against larval stage Echinococcus granulosus. Antimicrob. Agents Chemother. 58 (9), 5146–5154. 10.1128/AAC.02113-13 24936598 PMC4135819

[B22] Qi J. Sun C. Zebibula A. Zhang H. Kwok R. T. K. Zhao X. (2018). Real-time and high-resolution bioimaging with bright aggregation-induced emission dots in short-wave infrared region. Adv. Mater 30 (12), e1706856. 10.1002/adma.201706856 29341330

[B23] Qian M. B. Zhou X. N. (2018). Walk together to combat echinococcosis. Lancet Infect. Dis. 18 (9), 946. 10.1016/S1473-3099(18)30466-3 30152358

[B24] Qian Y. J. Ding W. Wu W. P. Bandikhuu A. Damdindorj T. Nyamdorj T. (2019). A path to cooperation between China and Mongolia towards the control of echinococcosis under the Belt and Road Initiative. Acta Trop. 195, 62–67. 10.1016/j.actatropica.2019.04.022 31009597

[B25] Shou K. Tang Y. Chen H. Chen S. Zhang L. Zhang A. (2018). Diketopyrrolopyrrole-based semiconducting polymer nanoparticles for *in vivo* second near-infrared window imaging and image-guided tumor surgery. Chem. Sci. 9 (12), 3105–3110. 10.1039/c8sc00206a 29732093 PMC5914543

[B26] Tamer G. Dündar D. Uzuner H. Baydemir C. (2015). Evaluation of immunochromatographic test for the detection of antibodies against Echinococcosis granulosus. Med. Sci. Monit. 21, 1219–1222. 10.12659/MSM.893155 25921809 PMC4427020

[B27] Tao Y. Wang S. Zhang X. Xia J. Guo J. Hou J. (2016). A rapid and convenient method for *in vivo* fluorescent imaging of protoscolices of echinococcus multilocularis. Korean J. Parasitol. 54 (2), 225–231. 10.3347/kjp.2016.54.2.225 27180584 PMC4870970

[B28] Torgerson P. R. Keller K. Magnotta M. Ragland N. (2010). The global burden of alveolar echinococcosis. PLoS Negl. Trop. Dis. 4 (6), e722. 10.1371/journal.pntd.0000722 20582310 PMC2889826

[B29] Wan H. Yue J. Zhu S. Uno T. Zhang X. Yang Q. (2018). A bright organic NIR-II nanofluorophore for three-dimensional imaging into biological tissues. Nat. Commun. 9 (1), 1171. 10.1038/s41467-018-03505-4 29563581 PMC5862886

[B30] Wang H. Li J. Guo B. Zhao L. Zhang Z. McManus D. P. (2016). *In vitro* culture of Echinococcus multilocularis producing protoscoleces and mouse infection with the cultured vesicles. Parasit. Vectors 9 (1), 411. 10.1186/s13071-016-1687-y 27457380 PMC4960901

[B31] Wang J. H. Jebbawi F. Bellanger A. P. Beldi G. Millon L. Gottstein B. (2018). Immunotherapy of alveolar echinococcosis via PD-1/PD-L1 immune checkpoint blockade in mice. Parasite Immunol. 40 (12), e12596. 10.1111/pim.12596 30315719 PMC6587932

[B32] Wang J. Y. Gao C. H. Steverding D. Wang X. Shi F. Yang Y. t. (2013). Differential diagnosis of cystic and alveolar echinococcosis using an immunochromatographic test based on the detection of specific antibodies. Parasitol. Res. 112 (10), 3627–3633. 10.1007/s00436-013-3550-9 23949310

[B33] Wen H. Vuitton L. Tuxun T. Li J. Vuitton D. A. Zhang W. (2019). Echinococcosis: Advances in the 21st century. Clin. Microbiol. Rev. 32 (2), e00075-18. 10.1128/CMR.00075-18 30760475 PMC6431127

[B34] Wu D. Xue D. Zhou J. Wang Y. Feng Z. Xu J. (2020). Extrahepatic cholangiography in near-infrared II window with the clinically approved fluorescence agent indocyanine green: A promising imaging technology for intraoperative diagnosis. Theranostics 10 (8), 3636–3651. 10.7150/thno.41127 32206113 PMC7069080

[B35] Xiong X. Li J. Gao D. Sheng Z. Zheng H. Liu W. (2022). Cell-membrane biomimetic indocyanine green liposomes for phototheranostics of echinococcosis. Biosens. (Basel) 12 (5), 311. 10.3390/bios12050311 PMC9138668 35624612

[B36] Yang X. Wang S. Zhang X. Ye C. (2022). Development of PVA-based microsphere as a potential embolization agent. Mater Sci. Eng. C Mater Biol. Appl. 135, 112677. 10.1016/j.msec.2022.112677 35581062

[B37] Yu X. Feng Z. Cai Z. Jiang M. Xue D. Zhu L. (2019). Deciphering of cerebrovasculatures via ICG-assisted NIR-II fluorescence microscopy. J. Mater Chem. B 7 (42), 6623–6629. 10.1039/c9tb01381d 31591622

[B38] Zebibula A. Nuernisha A. Xia L. Sun C. Yu X. Xue D. (2018). Ultrastable and biocompatible NIR-II quantum dots for functional bioimaging. Adv. Funct. Mster 9, 1703451. 10.1002/adfm.201703451

[B39] Zhang C. Shao Y. Yang S. Bi X. Li L. Wang H. (2017). T-Cell tolerance and exhaustion in the clearance of echinococcus multilocularis: Role of inoculum size in a quantitative hepatic experimental model. Sci. Rep. 7 (1), 11153. 10.1038/s41598-017-11703-1 28894272 PMC5593833

[B40] Zhang M. X. Yue J. Y. Cui R. Ma Z. Wan H. Wang F. (2018). Bright quantum dots emitting at ∼1,600 nm in the NIR-IIb window for deep tissue fluorescence imaging. Proc. Natl. Acad. Sci. U. S. A. 115 (26), 6590–6595. 10.1073/pnas.1806153115 29891702 PMC6042152

[B41] Zhang Z. Zhao J. Chen Z. Wu H. Wang S. (2023). A molybdenum-based nanoplatform with multienzyme mimicking capacities for oxidative stress-induced acute liver injury treatment. Inorg. Chem. Front. 10, 1305–1314. 10.1039/d2qi02318k

