# Supplementary material for: NIR-II fluorescence microscopic bioimaging for intrahepatic angiography and the early detection of Echinococcus multilocularis microlesions
Source: Front Bioeng Biotechnol. 2023 Apr 19;11:1157852. doi: 10.3389/fbioe.2023.1157852 (PMC10154522; doi:10.3389/fbioe.2023.1157852)
Supplement: Supplementary file 1 [file DataSheet2.docx]

Supplementary Material

NIR-II fluorescence microscopic bioimaging for intrahepatic angiography and the early detection of echinococcus multilocularis microlesions

Nuernisha Alifu^*†^, Ting Yan^†^, Jun Li, Lijun Zhu, Abudusalamu Aini, Siyiti Amuti, Juan Wu, Wenjing Qi, Gang Guo, Wenbao Zhang^*^, Xueliang Zhang^*^

*** Correspondence:**Nuernisha Alifu

nens_xjmu@126.com

Wenbao Zhang

wenbaozhang2013@xjmu.edu.cn

Xueliang Zhang

shuxue2456@126.com

*^†^ These authors contribute equally.*

**
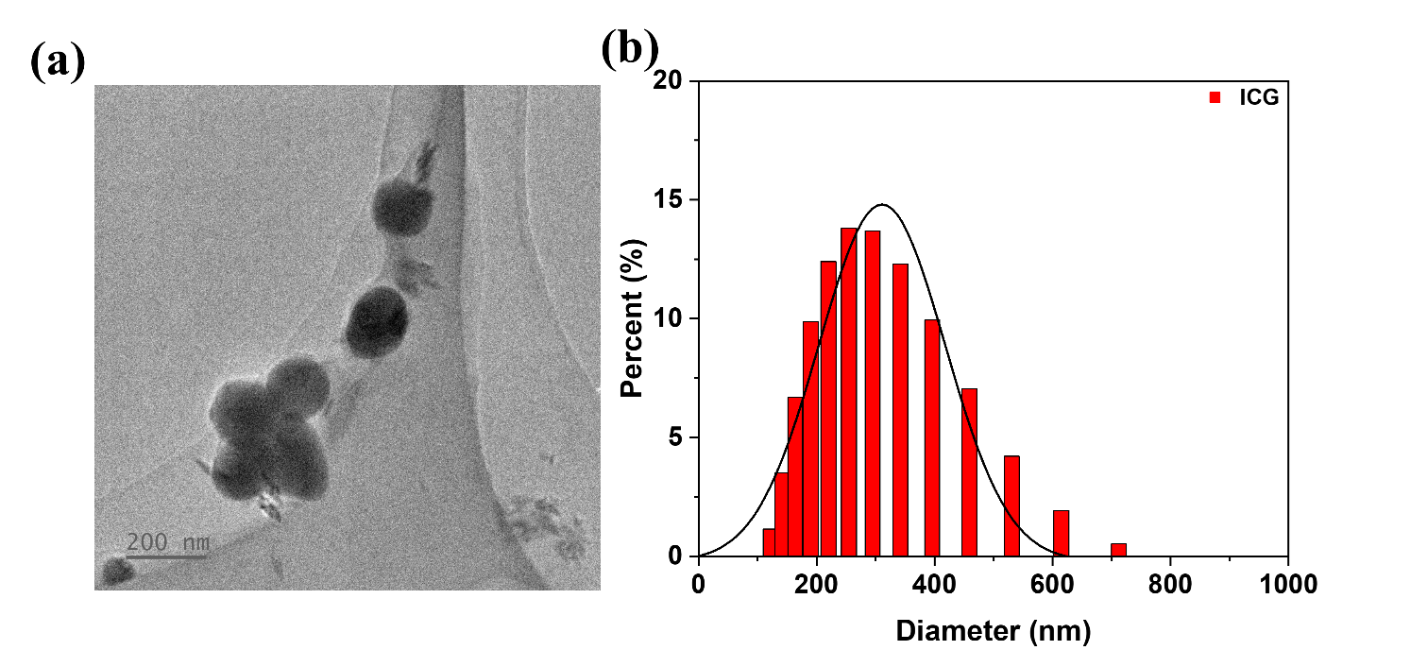
**

**Figure S1.** (a) TEM and (b) DLS image of ICG.

.**
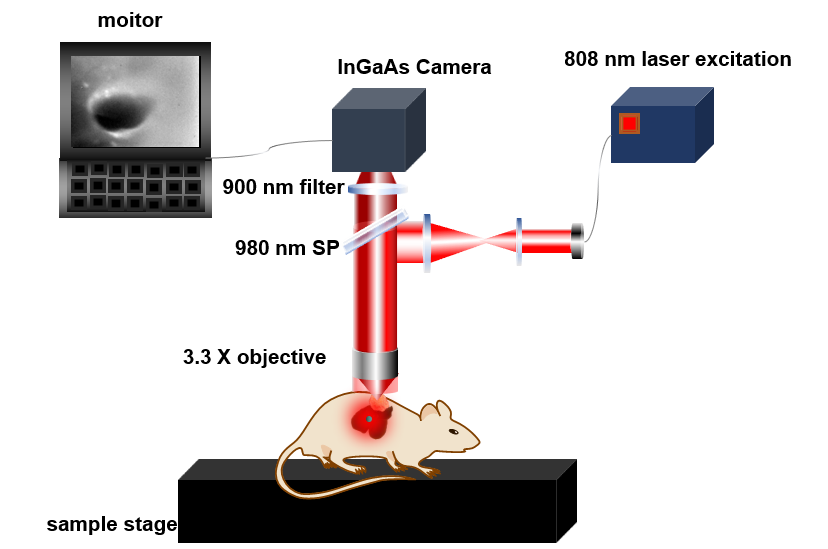
**

**Figure S2.** Schematic illustration of the upright NIR-II fluorescence microscopic imaging system.

**
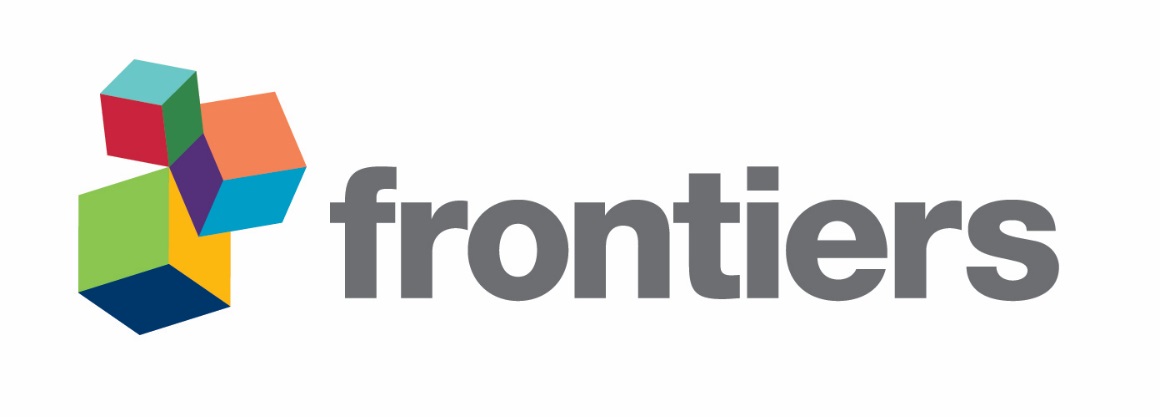
**
