# Supplementary material for: NIR-II fluorescence microscopic bioimaging for intrahepatic angiography and the early detection of Echinococcus multilocularis microlesions
Source: Front Bioeng Biotechnol. 2023 Apr 19;11:1157852. doi: 10.3389/fbioe.2023.1157852 (PMC10154522; doi:10.3389/fbioe.2023.1157852)

**Graphical Abstract Text**

NIR-II fluorescence microscopic imaging using ICG as a fluorescent nanoprobe was first time successfully applied to detecting HAE microlesions as negative targeting images in both early (1 month) and late (6 month) infection stages of HAE *in/ex vivo*. Clear NIR-II fluorescence microscopic intrahepatic angiography was achieved.

**Graphical Abstract Image**


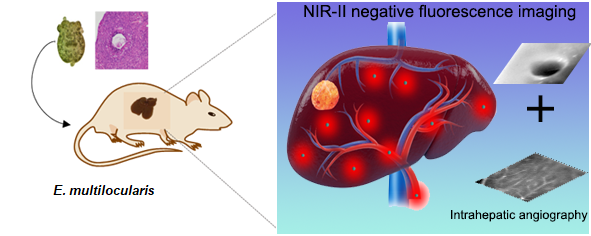

Supplement: Supplementary file 2 [file DataSheet1.docx]
